# Model-informed development of a cost-saving dosing regimen for enfortumab vedotin

**DOI:** 10.1007/s00280-025-04764-x

**Published:** 2025-02-25

**Authors:** Catharina JP Op ’t Hoog, Amy Rieborn, Dirk Jan AR Moes, Jeroen JMA Hendrikx, Michiel S. van der Heijden, Mira D. Franken, Tom van der Hulle, Michel van Kruchten, Annelieke ECAB Willemsen, Stijn LW Koolen, Emmy Boerrigter, Rob ter Heine

**Affiliations:** 1 Department of Pharmacy, Pharmacology & Toxicology - Research Institute for Medical Innovation, Radboud University Medical Center, (route 864), P.O. box 9101, Nijmegen, 6500 HB The Netherlands; 2 Department of Clinical Pharmacy & Toxicology, Leiden University Medical Centre, Leiden, The Netherlands; 3 Department of Pharmacy & Pharmacology, Netherlands Cancer Institute (NKI-AVL), Amsterdam, The Netherlands; 4 Department of Nuclear Medicine, Netherlands Cancer Institute (NKI-AVL), Amsterdam, The Netherlands; 5 Department of Medical Oncology, Netherlands Cancer Institute (NKI-AVL), Amsterdam, The Netherlands; 6 Department of Medical Oncology, Research Institute for Medical Innovation, Radboud University Medical Center, Nijmegen, The Netherlands; 7 Department of Medical Oncology, Leiden University Medical Centre, Leiden, The Netherlands; 8 Department of Medical Oncology, University Medical Center Groningen, Groningen, The Netherlands; 9 Department of Internal Medicine, Tergooi Medical Center, Hilversum, The Netherlands; 10 Department of Medical Oncology, Erasmus MC Cancer Institute, Erasmus University Medical Center, Rotterdam, the Netherlands; 11 Department of Clinical Pharmacy, Erasmus University Medical Center, Rotterdam, the Netherlands

**Keywords:** Enfortumab vedotin, Alternative dose, Model-informed drug development, Urethelial cancer, Antibody-drug conjugate

## Abstract

**Aim:**

Enfortumab vedotin is an antibody-drug conjugate (ADC) that has been approved for locally advanced or metastatic urothelial cancer, as monotherapy and in combination with pembrolizumab, and has shown significant benefit in progression-free survival and overall survival for these patients. The economic burden of enfortumab vedotin hampers widespread patient access. The aim of this study was to develop a model-informed alternative dosing regimen that results in equivalent drug exposure while reducing the costs and prevent drug spillage.

**Methods:**

Population pharmacokinetic modelling was used to simulate a dosing regimen leading to equivalent exposure by using the published population pharmacokinetic model in the registration reports. The alternative dosing regimen was based on weight-bands derived from the established non-linear relationship between body weight and systemic exposure, and the usage of whole vials based on fixed doses to prevent spillage. Equivalent exposure compared to the approved body weight-based dosing regimen was defined as conservative equivalent boundaries of 90–111% for the calculated geometric mean ratios (GMRs) of area under the concentration-time curve and trough concentration.

**Results:**

A weight-band based dosing regimen for each dose level of enfortumab vedotin was developed. The GMRs for all pharmacokinetic outcomes were within the predefined equivalence boundaries. In addition, a more even exposure distribution was observed across the body weight quartiles. The average costs savings across all dose levels and per weight-band were approximately 15%.

**Conclusion:**

The proposed alternative dosing regimen shows that drug costs and spillage of enfortumab vedotin can be reduced while maintaining an equivalent and more evenly distributed exposure in treated patients.

**Supplementary Information:**

The online version contains supplementary material available at 10.1007/s00280-025-04764-x.

## Introduction

Urothelial cancer (UC) cancer, usually occurring in the bladder, is an aggressive tumor type. Approximately 50% of all patients with muscle-invasive bladder cancer will develop metastatic disease. For decades, median overall survival has been less than 2 years for metastatic urothelial cancer (mUC) [1, 2], highlighting the low efficacy of previous available treatments. Management of advanced or metastatic disease previously consisted of cisplatin or carboplatin-combined with gemcitabine in first-line therapy followed by a programmed death receptor 1/programmed death ligand-1 (PD-1/PD-L1) inhibitor as maintenance therapy (avelumab) or in second-line therapy (pembrolizumab or atezolizumab) [3]. In recent years, the Food and Drug Administration (FDA) and European Medicines Agency (EMA) approved the antibody-drug conjugate enfortumab vedotin (EV) post-platinum and anti-PD-(L)1 checkpoint inhibitors for previously untreated advanced UC [4, 5]. These approvals changed the therapeutic landscape for these patients and significantly improved survival rates.

Enfortumab vedotin is composed of an anti-nectin-4 immunoglobulin G1 kappa monoclonal antibody conjugated to the small molecule microtubule-disrupting agent monomethyl auristatin E (MMAE), via a protease cleavable linker [4, 5]. Nectin-4, an adhesion protein highly expressed in epithelial cells of various malignant tumors including urothelial cancer, is targeted by enfortumab vedotin. Binding to nectin-4 releases MMAE in the target cells, leading to microtubule disruption, mitotic arrest and apoptotic cell death [6].

Enfortumab vedotin was first approved as third-line palliative treatment for patients with locally advanced or mUC who had received platinum-containing chemotherapy and disease progression during or after treatment with a PD-1/PD-L1 inhibitor [7, 8]. Data of the pivotal phase III trial (EV-301) demonstrated that enfortumab vedotin significantly improved overall survival (OS; median 12.9 vs. 9.0 months) compared to investigator-chosen chemotherapy [8]. More recently, the FDA and EMA approved enfortumab vedotin in combination with pembrolizumab as first-line treatment for patients with locally advanced UC or mUC. The phase III study (EV-302) demonstrated an impressive improvement with the combination therapy compared to platinum-based chemotherapy (median OS 31.5 vs. 16.1 months) [9].

Although enfortumab vedotin results in significant improvement in the outcomes of mUC, the drug comes with a hefty price tag, putting serious strains on healthcare budgets. In previous studies, enfortumab vedotin was not deemed as cost-effective in third-line treatment in the United States, United Kingdom and China at the current price point [10]. Additionally, enfortumab vedotin is dosed based on a linear body weight dosing algorithm (mg/kg) and the limited vial sizes (e.g., 20 and 30 mg) contribute to drug spillage. Body weight-based dosing can result in opening multiple vials, leading to partial usage and waste [11]. Furthermore, the linear mg/kg dosing paradigm suggests that the dose is already individualized based on body weight. However, as the true relationship between systemic exposure and body weight is less than proportional, this results in relative underdosing in patients with a low body weight and relative overdosing in patients with a high body weight [12]. This is reflected in the fact that high body weight is a risk factor for toxicity of enfortumab vedotin [13]. As more than half of patients experienced grade ≥ 3 toxicity (e.g. fatigue, peripheral sensory neuropathy, hyperglycemia or cutaneous toxicity), preventing relative overdosing may result in less toxicity while maintaining efficacy [8, 9, 14].

To reduce drug costs and minimize drug spillage multiple strategies have been proposed, including refund of unused drug amounts of single-use vials, the usage of a single-dose vial for multiple patients, and dose rounding of anticancer agents within margins of 10% [11, 15, 16]. Additionally, alternative dosage regimens such as prolongation of the dosing interval, or usage of fixeddoses could further optimize cancer treatment [17]. The FDA supports alternative dosage regimens as evidenced by their guideline for PD-1 and PD-L1 inhibitors, which allow the use of alternative dosage regimens based on population pharmacokinetic modelling and simulation without the need for clinical studies [18]. Additionally, the FDA guidance for Clinical Pharmacology Considerations for ADCs encourages evaluation of a broad dosage range, including multiple dosage levels and dosing regimens. It also suggests considering both fixed dose and weight-based dosing in early phase development [19]. Clinical studies of enfortumab vedotin have only investigated mg/kg dosing. Our study aims to propose an alternative weight-band based dosing regimen equivalent to the approved dosing regimen and to explore potential cost savings.

## Methods

### General approach

We performed a population pharmacokinetic simulation of the approved dosing regimen and an alternative dosing regimen using weight-bands, based on representative demographic data. In the approved dosing regimen, the initial dose starts at 1.25 mg/kg and is capped at 100 kg (125 mg). The dose may be reduced to 1 mg/kg, 0.75 mg/kg, or 0.5 mg/kg when a patient experiences toxicity. Enfortumab vedotin is administered on days 1, 8 and 15 of a 28-day cycle as monotherapy and on days 1 and 8 of a 21-day cycle in combination with pembrolizumab [4, 5, 20]. The alternative dosing regimen was developed based on the reported less than proportional relationship between body weight and systemic exposure [12, 21]. Doses were determined using weight-bands, ensuring that only complete vials were used to prevent spillage. The predicted pharmacokinetics of alternative dosing regimen for each individual weight-band had to result in equivalent exposure compared to the population mean pharmacokinetics at the approved dose and the associated pharmacokinetic variability should not significantly increase.

### Equivalence definition

The primary endpoints of our pharmacokinetic simulations were the area under the concentration-time curve (AUC) and the trough concentration (C_trough_) of enfortumab vedotin during the first cycle and at steady state. Since in our analysis no dose was increased or intervals were changed, no equivalence criteria were set for maximum concentrations (C_max_). The AUC and C_trough_ over the first cycle and at steady state of the approved and the alternative dosing regimen for the whole population are compared to assess equivalence. We used more conservative and strict bioequivalence boundaries of 90–111% since ADCs are considered to have a narrow therapeutic window. We thus declared bioequivalence if the geometric mean ratios (GMRs) of the pharmacokinetic endpoints of our study fell within the 0.9–1.1 boundaries [19, 22].

### Population pharmacokinetic modelling and simulation

For our population pharmacokinetic simulations, the population pharmacokinetic model for enfortumab vedotin as published in the EMA Assessment Report and FDA Multidisciplinary Review was used [4, 5]. This two-compartment model described the pharmacokinetics of the intact ADC and the unconjugated MMAE. Since the pharmacokinetics of the cytotoxic payload are described by a linear pharmacokinetic process, the pharmacokinetics of the ADC and the cytotoxic payload are directly proportional [4]. Therefore, the pharmacokinetics of the intact ADC are used as pharmacokinetic endpoints in the current study. A Monte Carlo simulation was performed using the non-linear mixed effects modeling (NONMEM) software package V7.5 (Icon, Dublin, Ireland). The NONMEM model code can be found in the supplementary material (S1). A fictional cohort of 500 European patients, derived from the European ICRP database in the PopGen virtual human population generator, was used to obtain representative demographic data [23].

First, the AUC and C_trough_ after the first cycle and at steady state were calculated from the simulated concentrations of the approved dose levels for enfortumab vedotin monotherapy (1.25 mg/kg, 1.0 mg/kg, 0.75 mg/kg and 0.5 mg/kg). Second, the alternative weight-band dosing regimen, based on the same administration days and a 28-day cycle, is calculated on weight-bands with the use of complete vials, while accounting for the allometric non-linear relationship between body size and pharmacokinetics. For all dosing regimens, the average quantity of drug used, based on the number of complete vials, was calculated for the alternative dosing regimen and compared to complete vials used based on the approved dose, under the assumption of complete spillage of a partially used vial. Fractional cost savings based on saved vials were calculated under the assumption of complete spillage of partially used vials as a “worst case scenario”. Average cost savings on a population level from a Dutch perspective were calculated for enfortumab vedotin monotherapy and combined with pembrolizumab, based on a median duration of treatment of 5 months (5 cycles) and 9.4 months (12 cycles) per patient, respectively [8, 9]. The average quantity of drug used for each dose was used in the calculation. Drug pricing of enfortumab vedotin per vial in the Netherlands was obtained through governmental data. Costs of Padcev^®^ in the Netherlands are € 698 for 20 mg and € 1,047 for 30 mg [24].

## Results

**Fig. 1:**
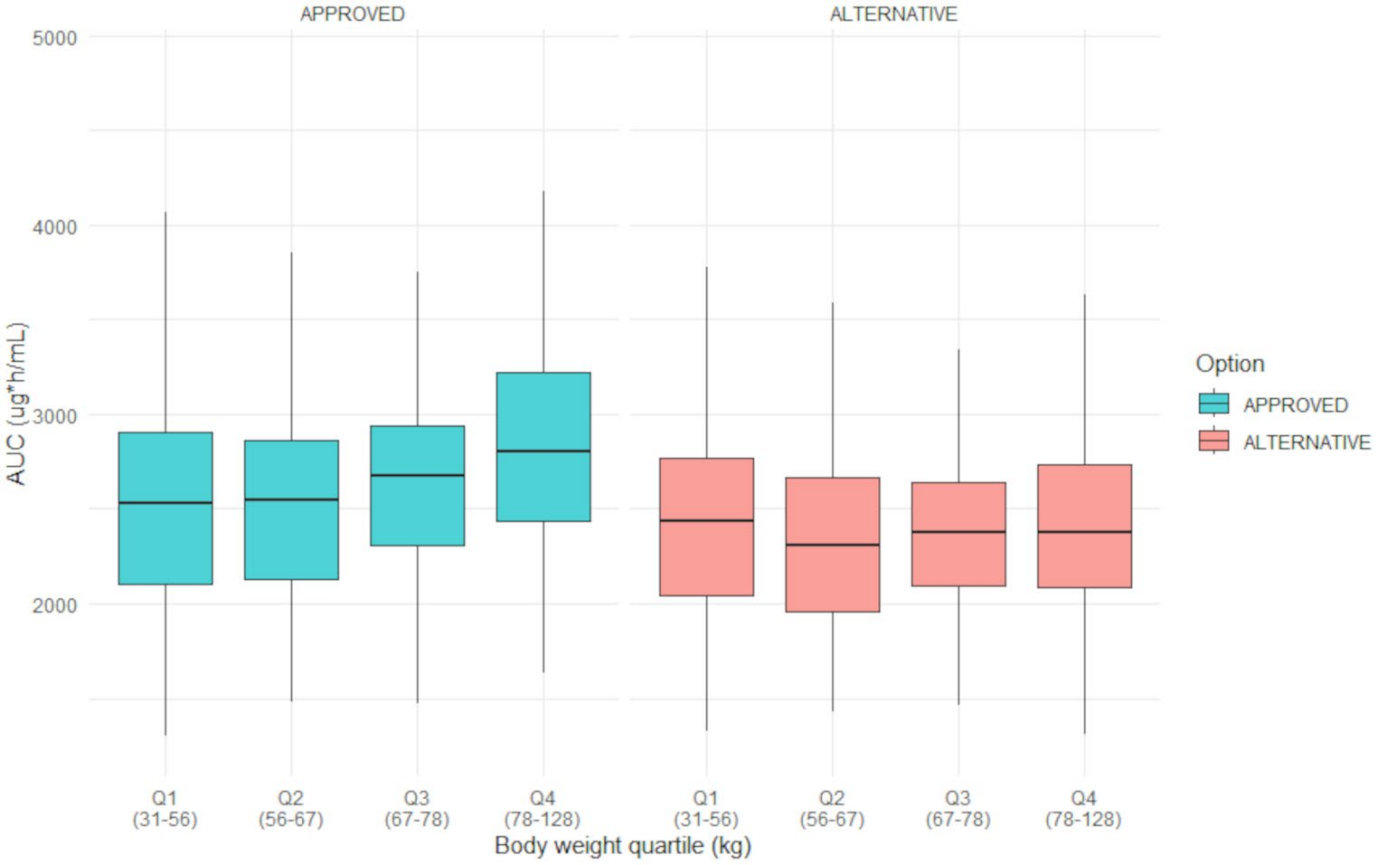
Boxplots of the predicted enfortumab vedotin steady state AUC per weight quartile of the approved and alternative dosing regimen

The alternative dosing regimen based on weight-band dosing are shown per dose level in Table 1. The results of the pharmacokinetic predictions and savings are presented in Table 2A-D for each dose level. The predicted geometric mean C_trough_ and AUC for the alternative dosing regimen were within the equivalence criteria of 90–111%, although slightly lower than the approved dosing regimen. Furthermore, the proposed weight-band dosing regimen does not increase the pharmacokinetic variability compared to body weight-based dosing, since difference in coefficients of variation (%CV) in AUC and C_trough_ between regimens is negligible. As shown in Fig. 1, a more even exposure distribution (AUC) across the body weight ranges can be observed in the alternative dosing regimen compared to the approved regimen. Additional visual representations of the exposure distribution at first cycle and at steady state for each dose level can be found in the supplementary material (Figures S1A–B to S4A-B). The median AUC and interquartile range (IQR) at first cycle and at steady state for the approved dosing regimen was 2558 mg*h/L (IQR 2162–2873 mg*h/L) and 2641 mg*h/L (IQR 2222–3005 mg*h/L), respectively. The median AUC and IQR at first cycle and at steady state for the alternative dosing regimen was 2304 mg*h/L (IQR 2014–2620 mg*h/L) and 2374 mg*h/L (IQR 2058–2714 mg*h/L), respectively. As observed in the Table 2A-D, on a population level approximately 15 to 20% savings in drug expenses can be realized while maintaining equivalent exposure. The average costs per treatment per patient of enfortumab vedotin monotherapy and in combination with pembrolizumab from a Dutch perspective are shown in Table 3.

**Table 1:** The alternative dosing regimen based on weight-band dosing for each dose level

Dose level	1.25 mg/kg	1.0 mg/kg	0.75 mg/kg	0.5 mg/kg
Weight band				
< 45 kg	50 mg	40 mg	30 mg	20 mg
45–55 kg	60 mg	50 mg	40 mg	20 mg
55–65 kg	70 mg	60 mg	40 mg	30 mg
65–75 kg	80 mg	60 mg	50 mg	30 mg
75–95 kg	90 mg	70 mg	50 mg	40 mg
> 95 kg	100 mg	80 mg	60 mg	40 mg

**Table 2A:** Results of pharmacokinetic simulation for the intact antibody-drug conjugate for dose level 1.25 mg/kg

Dosing regimen	AUC first cycle	C_trough_ first cycle	AUC steady state	C_trough_ steady state	Quantity of drug used for each dose	Quantity of drug saved for each dose per weight-band
	Geometric mean (%CV)	Geometric mean (%CV)	Geometric mean (%CV)	Geometric mean (%CV)	Average	Average
**Reference dose**	2510 mg*h/L (22%)	0.25 mg/L (99%)	2589 mg*h/L (23%)	0.29 mg/L (85%)	90.2 mg	
1.25 mg/kg						
**Alternative dose**	2284 mg*h/L (21%)	0.21 mg/L (99%)	2356 mg*h/L (22%)	0.26 mg/L (85%)	76.5 mg	8.5 mg
< 45 kg: 50 mg						
45–55 kg: 60 mg						7.2 mg
55–65 kg: 70 mg						10.6 mg
65–75 kg: 80 mg						13.9 mg
75–95 kg: 90 mg						17.9 mg
> 95 kg: 100 mg						30 mg
**Ratio Alternative dose/Reference dose**	0.910	0.910	0.910	0.910	0.848	

**Table 2B:** Results of pharmacokinetic simulation for the intact antibody-drug conjugate for dose level 1 mg/kg

Dosing regimen	AUC first cycle	C_trough_ first cycle	AUC steady state	C_trough_ steady state	Quantity of drug used for each dose	Quantity of drug saved for each dose per weight-band
	Geometric mean(%CV)	Geometric mean (%CV)	Geometric mean (%CV)	Geometric mean (%CV)	Average	Average
**Reference dose**	2008 mg*h/L (22%)	0.19 mg/L (99%)	2071 mg*h/L (23%)	0.23 mg/L (85%)	72.9 mg	
1.0 mg/kg						
**Alternative dose**	1825 mg*h/L (21%)	0.17 mg/L (99%)	1883 mg*h/L (22%)	0.21 mg/L (84%)	70.0 mg	8.8 mg
< 45 kg: 40 mg						
45–55 kg: 50 mg						5.3 mg
55–65 kg: 60 mg						5.7 mg
65–75 kg: 60 mg						15.7 mg
75–95 kg: 70 mg						17.1 mg
> 95 kg: 80 mg						20 mg
**Ratio Alternative dose/Reference dose**	0.909	0.909	0.909	0.909	0.836	

**Table 2C:** Results of pharmacokinetic simulation for the intact antibody-drug conjugate for dose level 0.75 mg/kg

Dosing regimen	AUC first cycle	C_trough_ first cycle	AUC steady state	C_trough_ steady state	Quantity of drug used for each dose	Quantity of drug saved for each dose per weight-band
	Geometric mean(%CV)	Geometric mean (%CV)	Geometric mean (%CV)	Geometric mean (%CV)	Average	Average
**Reference dose**	1506 mg*h/L (22%)	0.14 mg/L (99%)	1553 mg*h/L (23%)	0.17 mg/L (85%)	55.9 mg	
0.75 mg/kg						
**Alternative dose**	1365 mg*h/L (22%)	0.13 mg/L (100%)	1408 mg*h/L (23%)	0.16 mg/L (86%)	45.6 mg	8.8 mg
< 45 kg: 30 mg						
45–55 kg: 40 mg						1.8 mg
55–65 kg: 40 mg						10 mg
65–75 kg: 50 mg						8.1 mg
75–95 kg: 50 mg						16.6 mg
> 95 kg: 60 mg						20 mg
**Ratio Alternative dose/Reference dose**	0.906	0.906	0.906	0.906	0.814	

**Table 2D:** Results of pharmacokinetic simulation for the intact antibody-drug conjugate for dose level 0.5 mg/kg

Dosing regimen	AUC first cycle	C_trough_ first cycle	AUC steady state	C_trough_ steady state	Quantity of drug used for each dose	Quantity of drug saved for each dose per weight-band
	Geometric mean(%CV)	Geometric mean (%CV)	Geometric mean (%CV)	Geometric mean (%CV)	Average	Average
**Reference dose**	1004 mg*h/L (22%)	0.09 mg/L (99%)	1036 mg*h/L (23%)	0.11 mg/L (85%)	38.9 mg	
0.5 mg/kg						
**Alternative dose**	911 mg*h/L (23%)	0.08 mg/L (99%)	939 mg*h/L (24%)	0.10 mg/L (85%)	31.0 mg	8.8 mg
< 45 kg: 20 mg						
45–55 kg: 20 mg						10 mg
55–65 kg: 30 mg						5.7 mg
65–75 kg: 30 mg						10 mg
75–95 kg: 40 mg						6.1 mg
> 95 kg: 40 mg						10 mg
**Ratio Alternative dose/Reference dose**	0.907	0.907	0.907	0.907	0.796	

**Table 3:** Average costs per treatment per patient on a population level for enfortumab vedotin monotherapy and in combination with pembrolizumab from a Dutch perspective

Dose level	Approved dosing regimen		Alternative dosing regimen		Average savings
	Enfortumab vedotin monotherapy	Enfortumab vedotin combination therapy	Enfortumab vedotin monotherapy	Enfortumab vedotin combination therapy	
	Average costs per treatment per patient	Average costs per treatment per patient	Average costs per treatment per patient	Average costs per treatment per patient	
**1.25 mg/kg**	€ 47,218.35	€ 75,549.36	€ 40,046.60	€ 64,074.56	15%
**1.0 mg/kg**	€ 38,162.06	€ 61,059.29	€ 36,643.95	€ 58,630.32	4%
**0.75 mg/kg**	€ 29,262.81	€ 46,820.50	€ 23,870.92	€ 38,193.47	18%
**0.5 mg/kg**	€ 20,363.57	€ 32,581.71	€ 16,228.04	€ 25,964.86	20%

## Discussion

Our investigation shows that an alternative dosing regimen based on weight-bands for enfortumab vedotin will reduce drug spillage and reduce drug expenses. Up to 20% of drug costs can be saved while maintaining an equivalent exposure for the population across the weight range. Moreover, the observed exposure appears more evenly distributed across the bodyweight range in the alternative regimen compared to the approved regimen. The pharmacokinetic variability with this weight-band dosing regimen does not increase and shows that variability does not increase with weightband dosing compared to linear mg/kg dosing.

The predicted GMRs of the AUC and C_trough_ of the alternative dosing regimen lie within the equivalence boundaries for drugs with a narrow therapeutic window. In our analysis, the reduction for all calculated AUC and C_trough_ for the whole population in the alternative dosing regimen were less than 10%. In the exposure-response analysis performed by the manufacturer, no statistically significant exposure-efficacy relationship was found for OS. However, a positive trend was observed for the response rate for all exposure quartiles. In this analysis, the average concentrations (C_avg_) was used as the exposure metric [25]. Although this metric can be linked to AUC, other metrics, such as C_max_ and C_trough_, were not explored. Additionally, exposure levels of MMAE based on C_avg_ negatively correlated with OS [25]. Due to the absence of a significant exposure-efficacy relationship for OS and the observed response across the studied dosing range of 0.5 mg/kg to 1.25 mg/kg, the slight reduction in exposure is unlikely to impact efficacy [14, 25–27]. Moreover, in the exposure-response analysis a significant exposure-toxicity relationship was observed. Patients in higher body weight quartiles had a relatively greater exposure, leading to an increased safety risk [14, 25, 28]. Specifically, enfortumab vedotin-induced peripheral neuropathy is a common adverse event leading to potentially permanent symptoms and disability, and which could significantly impact treatment adherence and interfere with long-term treatment outcomes [14, 28]. In our analysis, the proposed alternative dosing regimen resulted in a slight reduction in exposure for the population and a more even distribution across all body weight quartiles, which may improve safety and lower the risk of adverse events. This also implies some nuance to the conclusion by the manufacturer that a weight-based dosing regimen suggests a smaller variability in exposure compared to fixed dosing. In their exposure-response analysis, the weight-based dosing regimen was compared to a fixed-dose of 95 mg (based on an average body weight of 75 kg) [25], as opposed to the proposed rounded weight-band dosing regimen in our study. In addition to the accordance of the exposure metrics of the alternative dosing regimen with the FDA guideline for PD-1/PD-L1 inhibitors, the used equivalence boundaries of 90–111% in our analysis are conservative. Since our analysis relied on the pharmacokinetic model provided by the manufacturer and employed conservative criteria for equivalence, we propose that this dosing regimen can be readily implemented in clinical practice. Analogous dosing regimens developed through modelling and simulation for PD-1/PD-L1 inhibitors are endorsed by FDA guidelines, supporting their implementation [18].

Major advances in anticancer therapy developments with a hefty price tag support the need for cost reduction. The ADCs are an emerging class of potent anticancer drugs that provide highly effective therapy advances in the treatment of hematologic and solid malignancies. Coupled with these advances are economic challenges related to the ability to pay for these therapies together with the growing number of eligible patients and the expansion of approved indications. Enfortumab vedotin is no exception. In the Netherlands, total costs of enfortumab vedotin monotherapy were over €2,300,000 in 2023 [29]. Although the results of the phase 3 study in combination with pembrolizumab are impressive, the addition of pembrolizumab will even further increase drug expenses [9]. Furthermore, the shift to first-line therapy will increase the number of eligible patients. It is estimated that in the Netherlands alone around 1,200 patients with advanced or metastatic UC per year will be eligible for the first-line combination therapy [30]. Based on a median 12 cycles per patient [9], the total costs per patient are estimated to be € 152,411.04 and in total more than € 182,000,000 per year, from a Dutch perspective, with long-term responders having even a more pronounced effect on costs [24, 30]. This emphasizes the need to optimize the cost-effectiveness of enfortumab vedotin treatment and further reduce the drug costs without compromising effective exposure. In our analysis, a reduction at the 1.25 mg/kg dose level of 15% could be achieved. This could lead to cost savings up to approximately € 22,000 per patient based on whole vials, and a reduction of € 27.000.000 each year from a Dutch perspective. When conducting a cost-effectiveness analysis based on our proposed alternative dosing regimen, all factors remain the same with the exception of the drug costs. Since drug costs will be lower, the alternative dosing regimen will have a higher probability to be cost-effective. Since drug prices of enfortumab vedotin and the variation in body weight may vary across countries, the reduction in costs could potentially be even greater. For example, body weight in the United States of America is generally higher, thus the cost savings of the alternative dosing regimen on a population level will be greater with an increasing body weight [31].

In addition to the cost savings, using rounded doses based on weight-bands and the use of whole vials can have added benefit in reducing drug spillage compared to individual weight-based dosing with partially used vials. In clinical practice, it may happen that a drug has already been prepared, but the patient is not able to receive the drug. In the case of individual weight-based dosing, the prepared infusion bag will be thrown away, whereas with a rounded dose, the infusion bag could potentially be used for another patient with a similar body weight.

In conclusion, this study shows that drug costs and drug spillage of enfortumab vedotin can be reduced by using a weight-based dosing regimen while maintaining an equivalent exposure and achieving a more evenly distributed exposure across the body weight range. Although potential cost reduction and waste avoidance begins with the manufacturer, the possibilities that are provided with alternative dosing regimens should be further explored by both clinical healthcare providers and government. Since the costs of enfortumab vedotin together with the growing number of patients that are eligible for combination therapy with pembrolizumab are substantial, implementation of the presented alternative dosing regimen should be considered.

## Electronic supplementary material

Below is the link to the electronic supplementary material.

Supplementary Material 1

## Data Availability

Data are available on reasonable request from the authors.Code AvailabilityCode available in the supplemental material.
