# Supplementary material for: Model-informed development of a cost-saving dosing regimen for enfortumab vedotin
Source: Cancer Chemother Pharmacol. 2025 Feb 25;95(1):36. doi: 10.1007/s00280-025-04764-x (PMC11860999; doi:10.1007/s00280-025-04764-x)

Model-informed development of a cost-saving dosing regimen for enfortumab vedotin

Catharina JP Op ’t Hoog^1^, Amy Rieborn^2^, Dirk Jan AR Moes^2^, Jeroen JMA Hendrikx^3^, Michel S van der Heijden^4^, Mira D Franken^5^, Tom van der Hulle^6^, Michel van Kruchten^7^, Annelieke ECAB Willemsen^8^, Stijn LW Koolen^9^, Emmy Boerrigter^1^, Rob ter Heine^1,$^

^1^ Department of Pharmacy, Research Institute for Medical Innovation, Radboud University Medical Center, Nijmegen, The Netherlands.

^2^ Department of Clinical Pharmacy & Toxicology, Leiden University Medical Centre, Leiden, The Netherlands.

^3^ Department of Pharmacy & Pharmacology, Netherlands Cancer Institute (NKI-AVL), Amsterdam, The Netherlands; Department of Nuclear Medicine, Netherlands Cancer Institute (NKI-AVL), Amsterdam, The Netherlands.

^4^ Department of Medical Oncology, Netherlands Cancer Institute (NKI-AVL), Amsterdam, The Netherlands

^5^ Department of Medical Oncology, Research Institute for Medical Innovation, Radboud University Medical Center, Nijmegen, The Netherlands.

^6^ Department of Medical Oncology, Leiden University Medical Centre, Leiden, The Netherlands.

^7^ Department of Medical Oncology, University Medical Center Groningen, Groningen, The Netherlands.

^8^ Department of Internal Medicine, Tergooi Medical Center, Hilversum, The Netherlands.

^9^ Department of Medical Oncology, Erasmus MC Cancer Institute, Erasmus University Medical Center, Rotterdam, the Netherlands; Department of Clinical Pharmacy, Erasmus University Medical Center, Rotterdam, the Netherlands.

**^$^Corresponding Author**

Dr. Rob ter Heine

Radboudumc, Research Institute for Medical Innovation

Department of Pharmacy (route 864)

P.O. box 9101, 6500 HB Nijmegen, The Netherlands

Tel: +31 24 361 11 11

[R.terHeine@radboudumc.nl](mailto:R.terHeine@radboudumc.nl)

# Supplementary material

**S1 NONMEM model control stream**

$PROB ENFORTUMAB VEDOTIN

$INPUT ID AGE SEX WT HEIGHT TIMEDAYS TIME AMT CMT MDV EVID DV RATE

$DATA xxx IGNORE=@

$SUBROUTINE ADVAN13 TOL=5

$MODEL

COMP=(CENTRALADC) ;1

COMP=(PERIADC) ;2

COMP=(PERI2ADC) ;3

COMP=(AUC1ADC) ;4

COMP=(AUCSSADC) ;5

$PK

D1=0.5 ; INFUSION IN 30 MINUTES ON DAY 1,8,15 OF 28 DAY CYCLE

ALLOCL=(WT/70)**0.761

ALLOV=(WT/70)**0.710

; --- ADC

CL=THETA(1)*ALLOCL*EXP(ETA(1))

V1=THETA(2)*ALLOV*EXP(ETA(2))

Q2=THETA(3)*ALLOCL

V2=THETA(4)*ALLOV

Q3=THETA(5)*ALLOCL

V3=THETA(6)*ALLOV*EXP(ETA(3))

S1=V1

K10=CL/V1

K12=Q2/V1

K21=Q2/V2

K13=Q3/V1

K31=Q3/V3

$DES

; --- PK

DADT(1)=-K10*A(1)-K12*A(1)-K13*A(1)+K21*A(2)+K31*A(3)

DADT(2)=-K21*A(2)+K12*A(1)

DADT(3)=-K31*A(3)+K13*A(1)

C1=A(1)/V1

FLA1=0

FLASS=0

IF (T.LT.672) FLA1=1 ; FIRST 28 DAYS

IF (T.GE.6048) FLASS=1 ; DAYS 252-280

DADT(4)=FLA1*C1 ; AUC1

DADT(5)=FLASS*C1 ; AUCSS

$ERROR

IPRED=F

IF (CMT.EQ.1) Y=F+F*ERR(1)

AUCFIRST=A(4)

AUCSS=A(5)

$THETA

0.0992 ; 1 CL L/H

3.52 ; 2 V1 L

0.00220 ; 3 Q2 L/H

2.49 ; 4 V2 L

0.0378 ; 5 Q3 L/H

2.68 ; 6 V3 L

$OMEGA

0.05108 ; IIV CL

0.065 ; IIV V1

0.183 ; IIV V3

$SIGMA 0 FIX ; PROP ERR

$SIMULATION ONLYSIM SUBPROBLEMS=1 (22512) (174292)

Figure S1A and S1B: exposure distribution of enfortumab vedotin at steady state and first cycle for dose level 1.25 mg/kg


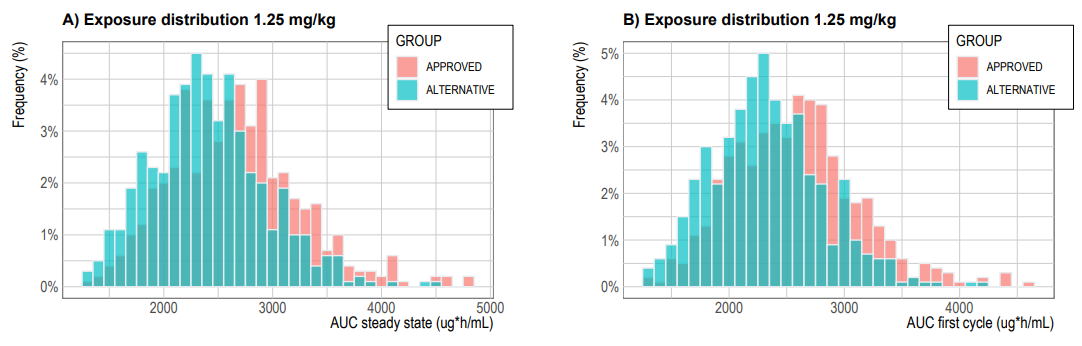


Figure S2A and S2B: exposure distribution of enfortumab vedotin at steady state and first cycle for dose level 1.0 mg/kg


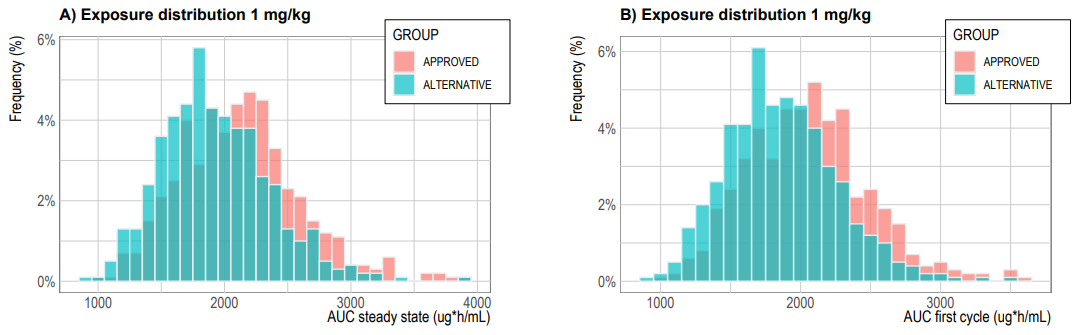


Figure S3A and S3B: exposure distribution of enfortumab vedotin at steady state and first cycle for dose level 0.75 mg/kg


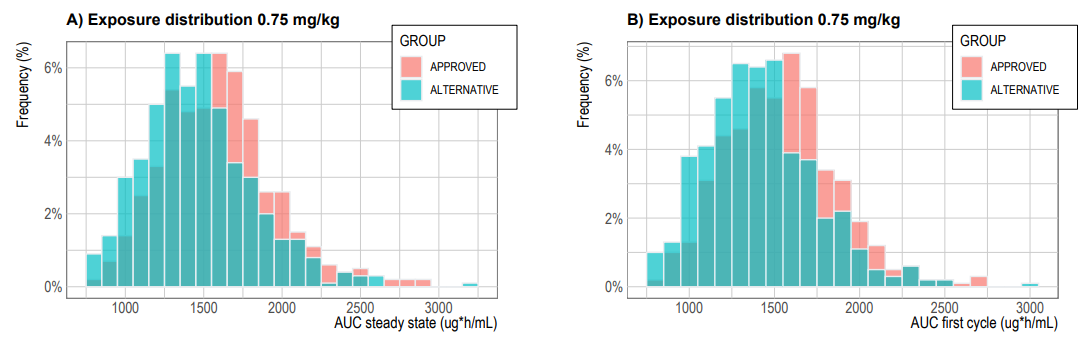


Figure S4A and S4B: exposure distribution of enfortumab vedotin at steady state and first cycle for dose level 0.5 mg/kg


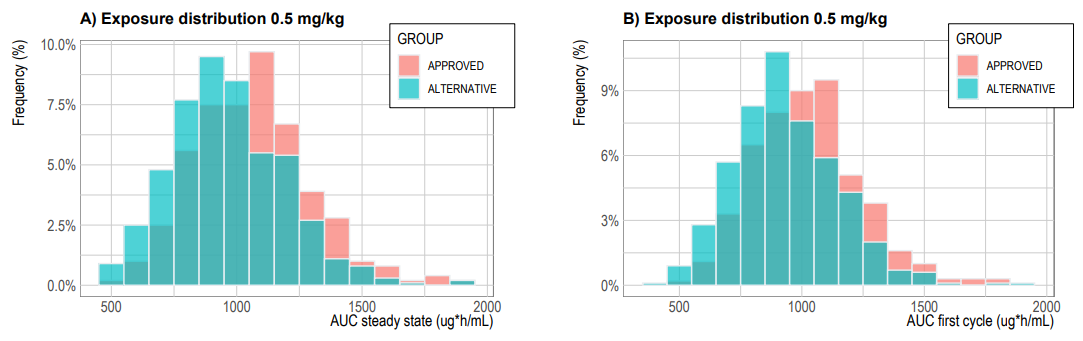

Supplement: Supplementary file 1 — Supplementary Material 1 [file 280_2025_4764_MOESM1_ESM.docx]
